# Supplementary figures and images for: Reprogramming and redifferentiation of mucosal-associated invariant T cells reveal tumor inhibitory activity
Source: eLife. 2022 Apr 5;11:e70848. doi: 10.7554/eLife.70848 (PMC8983048; doi:10.7554/eLife.70848)

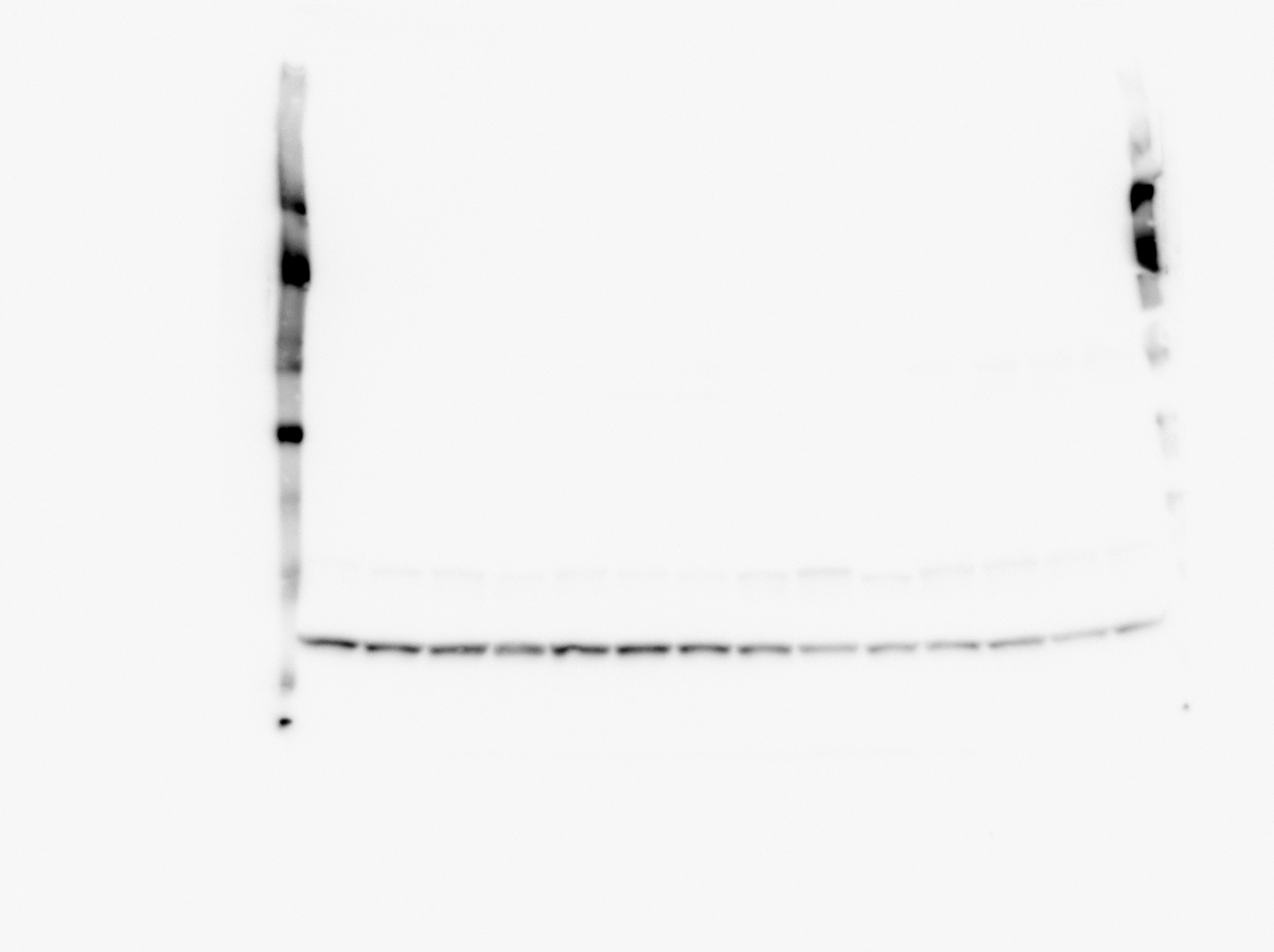

Supplement: Figure 1—source data 2. [file elife-70848-fig1-data2.zip › Source data Fig1/Fig1I b_actin original.tif]

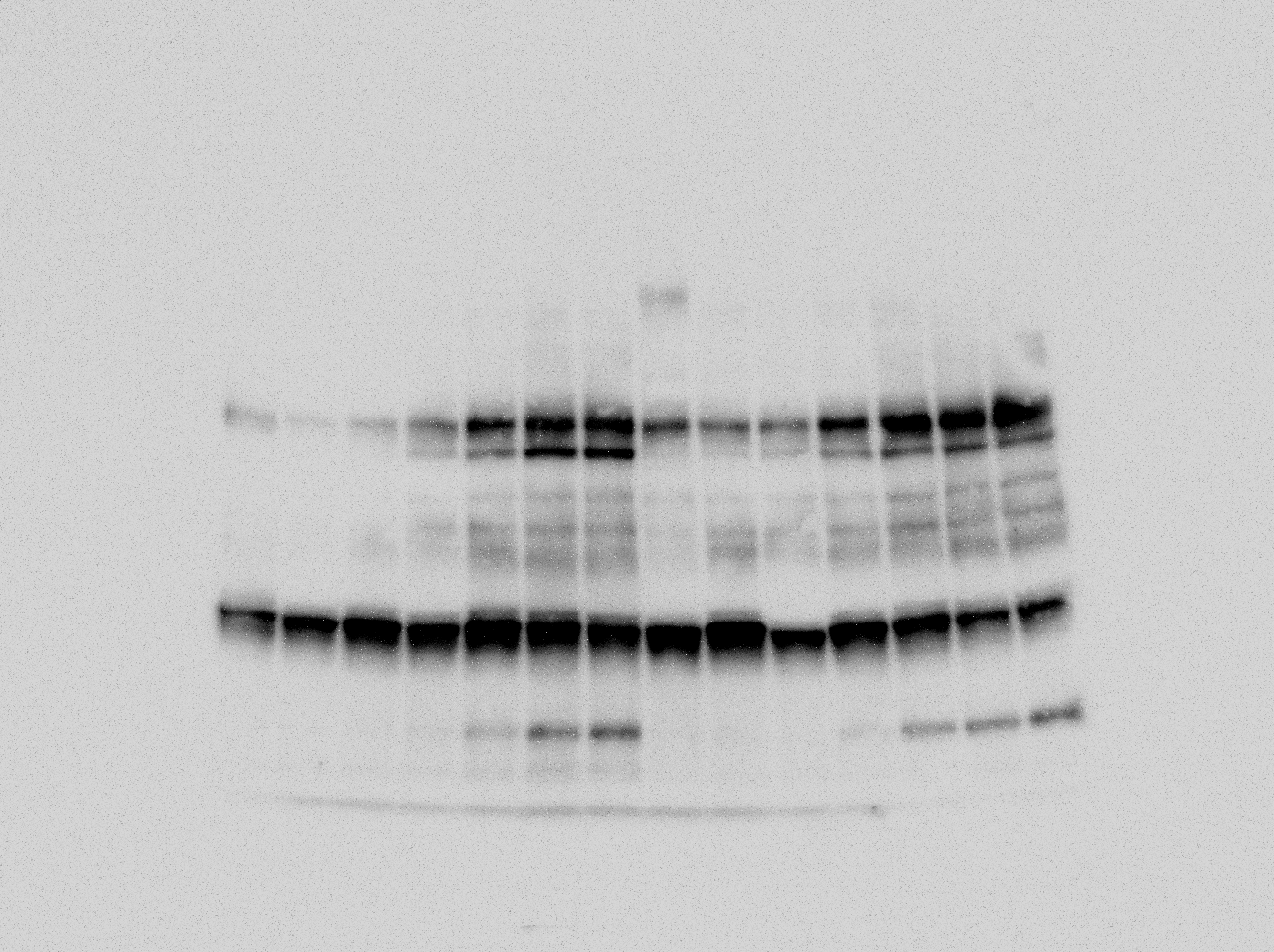

Supplement: Figure 1—source data 2. [file elife-70848-fig1-data2.zip › Source data Fig1/Fig1G&I PY99 original.tiff]

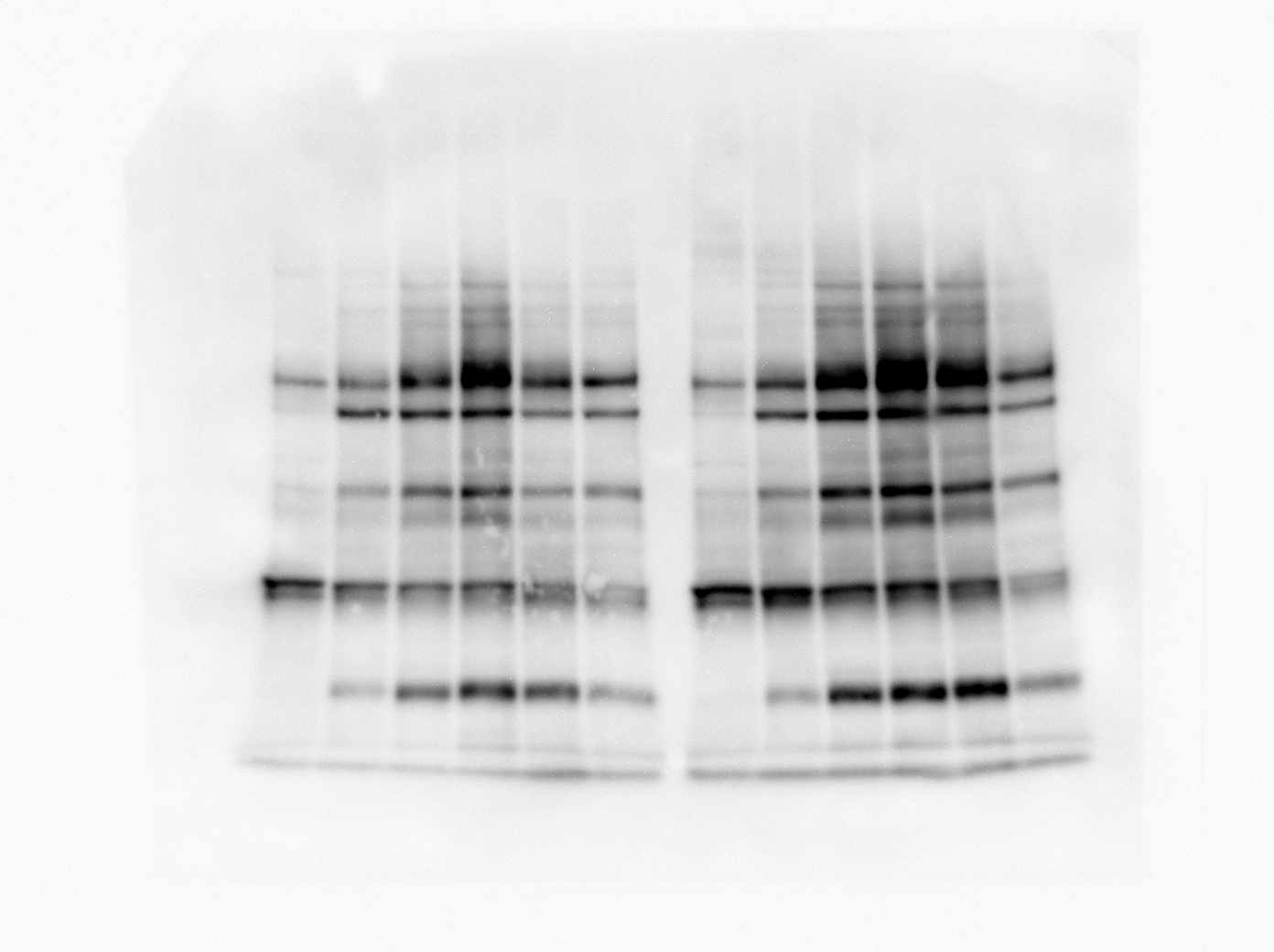

Supplement: Figure 1—source data 2. [file elife-70848-fig1-data2.zip › Source data Fig1/Fig1H original.tiff]

## Slide 1
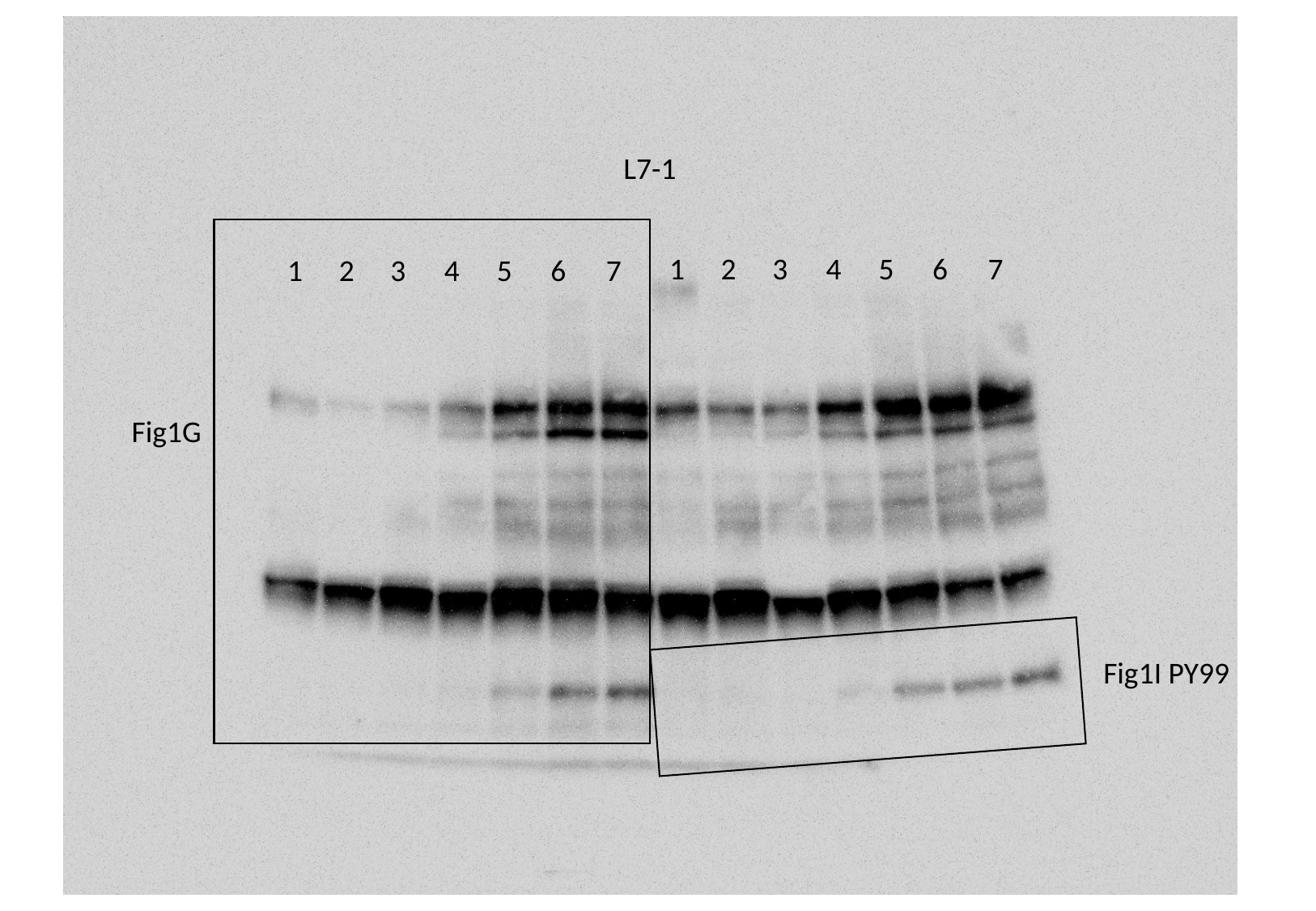

L7-1
1
2
3
4
5
6
7
1
2
3
4
5
6
7
Fig1G
Fig1I PY99

## Slide 2
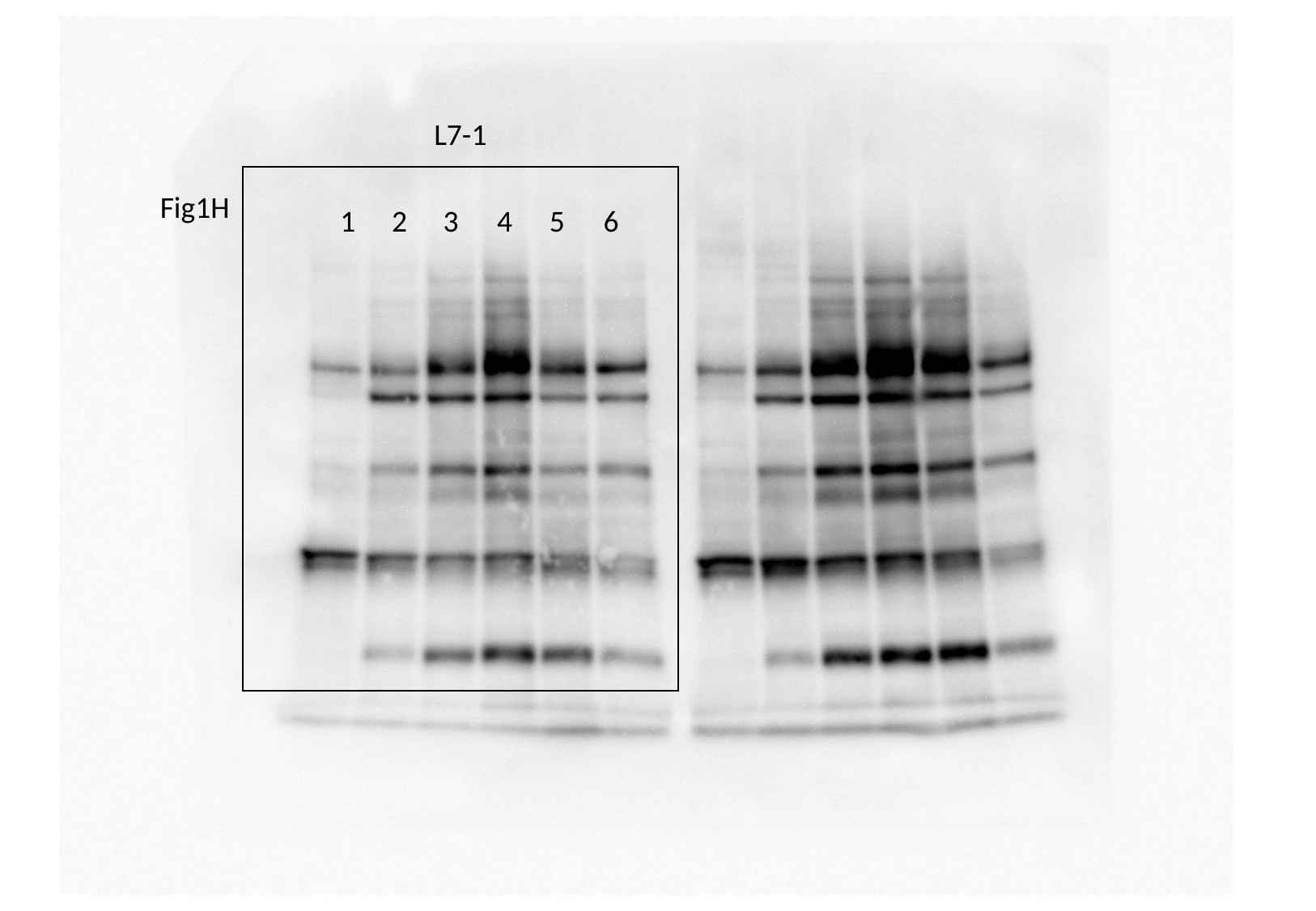

L7-1
Fig1H
1
2
3
4
5
6

## Slide 3
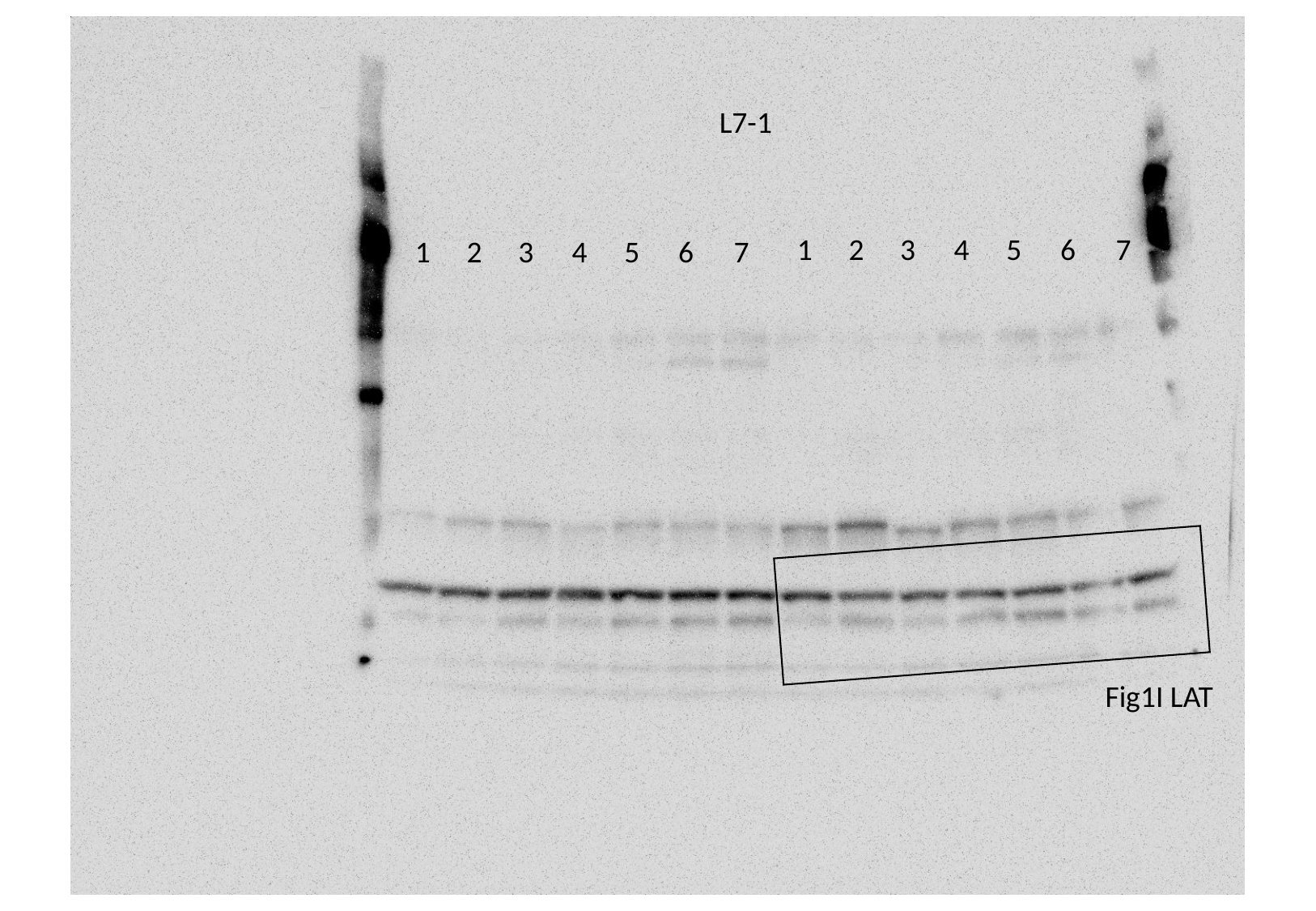

L7-1
1
2
3
4
5
6
7
1
2
3
4
5
6
7
Fig1I LAT

## Slide 4
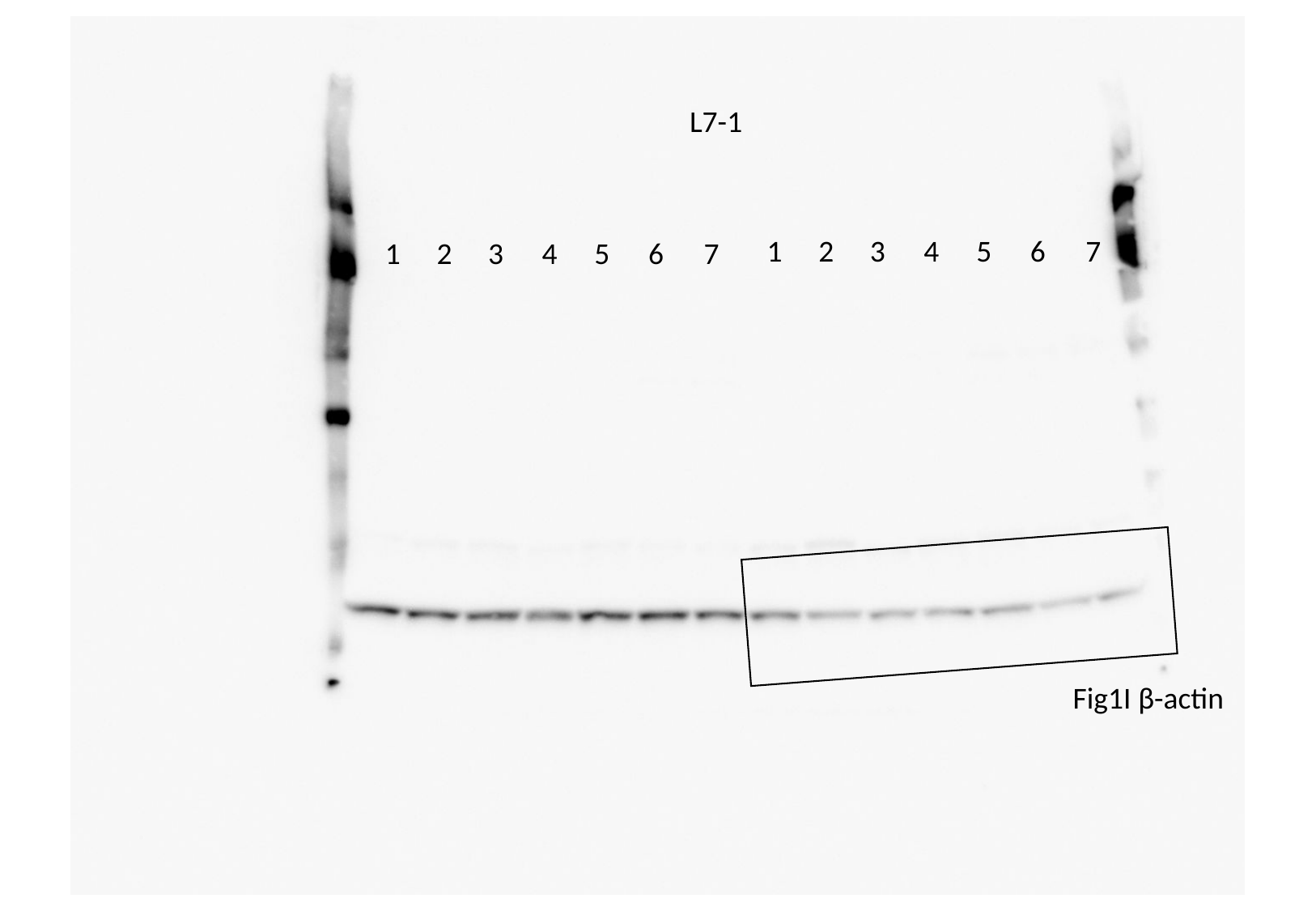

L7-1
1
2
3
4
5
6
7
1
2
3
4
5
6
7
Fig1I β-actin

## Slide 5
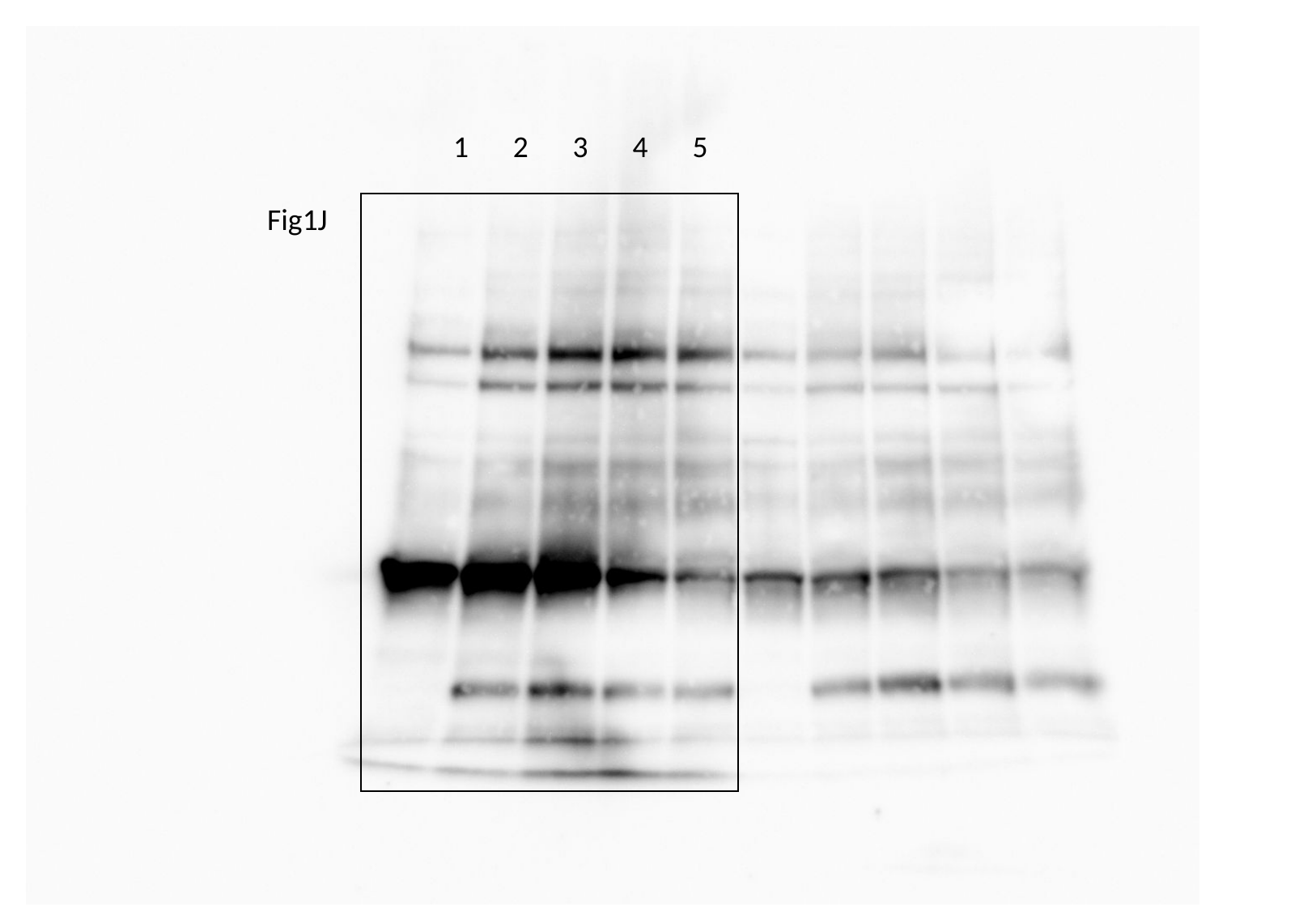

1
2
3
4
5
Fig1J

Supplement: Figure 1—source data 2. [file elife-70848-fig1-data2.zip › Source data Fig1/Fig1 source data 2.pptx]

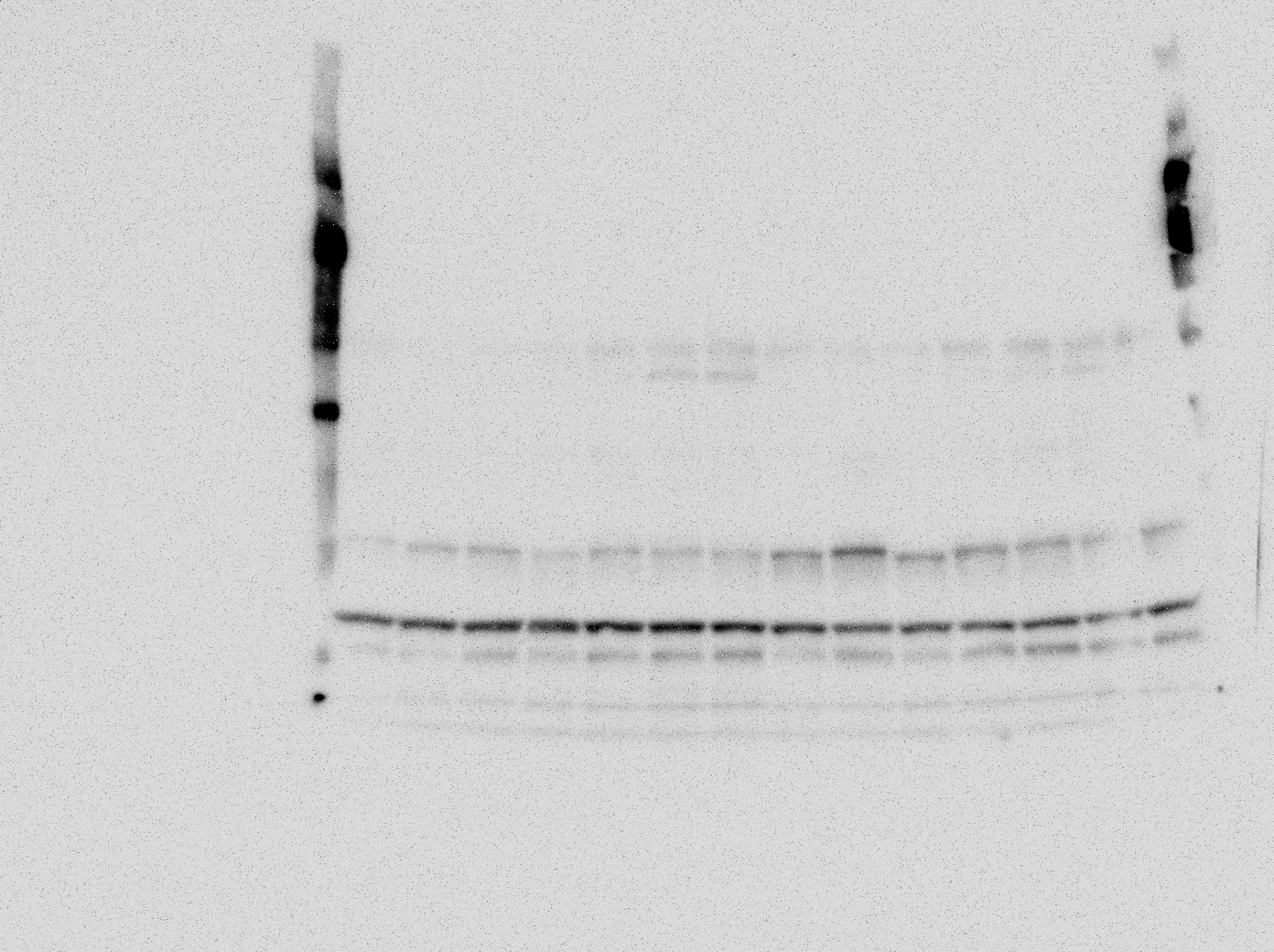

Supplement: Figure 1—source data 2. [file elife-70848-fig1-data2.zip › Source data Fig1/Fig1I LAT original.tif]

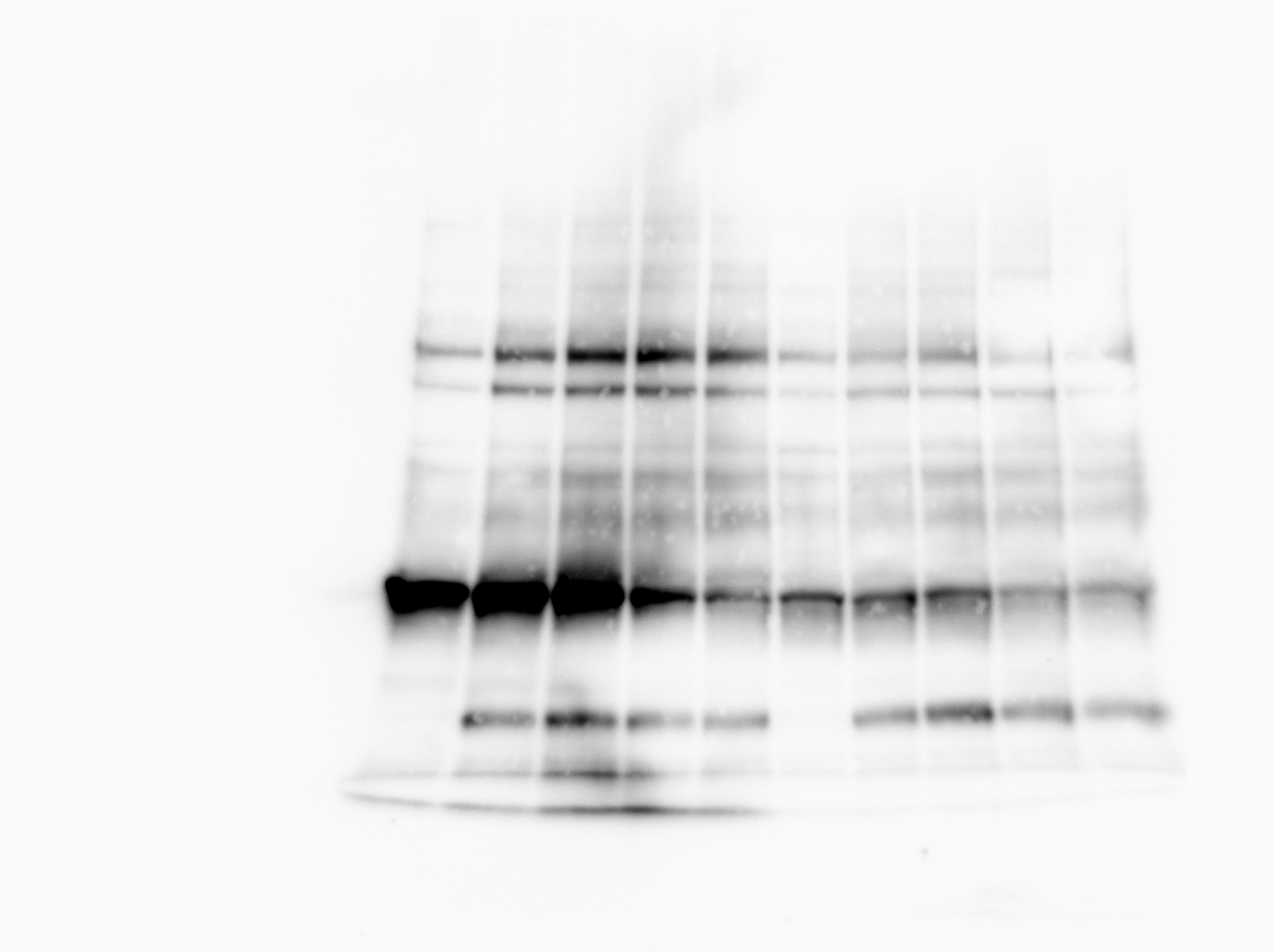

Supplement: Figure 1—source data 2. [file elife-70848-fig1-data2.zip › Source data Fig1/Fig1J original.tiff]
